# High resolution multi-parametric probabilistic *in vivo* atlas of dorsolateral nigral hyperintensity via 7 T MRI

**DOI:** 10.1038/s41597-025-05325-w

**Published:** 2025-06-07

**Authors:** Marta Lancione, Graziella Donatelli, Gianmichele Migaleddu, Matteo Cencini, Paolo Bosco, Mauro Costagli, Roberto Ceravolo, Mirco Cosottini, Michela Tosetti, Laura Biagi

**Affiliations:** 1 IRCCS Stella Maris Foundation, Pisa, Italy; 2 IMAGO7 Foundation, Pisa, Italy; 3 Neuroradiology Unit, Department of Translational Research on New Technologies in Medicine and Surgery, University of Pisa, Pisa, Italy; 4 Neuroradiology Unit, Azienda Ospedaliero-Universitaria Pisana, Pisa, Italy; 5 Department of Neuroscience, Rehabilitation, Ophthalmology, Genetics, Maternal and Child Health (DINOGMI), University of Genoa, Genoa, Italy; 6 IRCCS Ospedale Policlinico San Martino, Genoa, Italy; 7 Neurology Unit, Department of Clinical and Experimental Medicine, University of Pisa, Pisa, Italy

**Keywords:** Medical research, Brain

## Abstract

The role of Nigrosome 1 (N1) in neurodegeneration and motor disorders, particularly in Parkinson’s disease (PD), is increasingly recognized. The study of this region using quantitative measures, such as iron quantification through Quantitative Susceptibility Mapping (QSM), can provide enlightening insights into some pathological features of these diseases representing important biomarkers. However, the small size and the vanishing contrast with respect to the surrounding substantia nigra in PD patients make the segmentation of N1 challenging. For this reason, we provide a probabilistic atlas of the N1 portion corresponding to the swallow-tail hyperintensity, hereafter referred to as the Dorsolateral Nigral Hyperintensity (DNH), created on a high-resolution multi-parametric template from T1-weighted, T2*-weighted, and QSM images acquired *in vivo* at 7 T. The atlas also includes quantitative T2* and R2* templates and is provided in the MNI standard space. It aims to facilitate the study of N1, avoiding operator-dependent biases in segmentations, and allowing the standardisation of the quantitative assessment.

## Background & Summary

Nigrosome 1 (N1) is a small area of the substantia nigra (SN) defined at pathology as the largest calbindin-poor zone of the SN pars compacta^1,2^ and corresponds to the lateral tier of the pars compacta ventralis^1^. It has a critical role in the regulation of movement and in the pathophysiology of motor disorders, in particular Parkinson’s disease (PD). In PD patients, indeed, it is severely affected by the processes of degeneration of the dopaminergic neurons and iron accumulation^2,3^.

The exact anatomical-radiological correlation of N1 is still partly elusive. N1 was considered for a long time as the anatomical counterpart of the swallow-tail sign that is, in healthy subjects, the T2* hyperintense ovoid shaped area located in the dorsolateral part of the SN pars compacta^4,5^. This concept has been recently redefined, and the swallow-tail sign was shown to only partly overlap with N1^6^. However, further studies are needed to definitively define the correspondence between the radiological sign and the underlying anatomical structures. To acknowledge that the swallow-tail sign may only partially overlap to N1, hereinafter we will refer to it as the Dorsolateral Nigral Hyperintensity (DNH).

From the radiological standpoint, DNH has been proven to be lost (and the SN pars compacta ventralis to be homogeneously hypointense) in most idiopathic PD patients imaged with both 3 T and 7 T Magnetic Resonance Imaging (MRI)^7^; this is considered a biomarker of PD^4,5,8^ and can be also detected in the prodromal stages^8–10^. Moreover, N1 degeneration and DNH loss were also reported in other neurodegenerative disorders such as Lewy body dementia^11^, multiple system atrophy^12^, and amyotrophic lateral sclerosis^13^.

Despite the clinical importance of this region and the great number of studies on SN iron deposition in PD^14^, a quantitative MRI-based assessment specifically focused on DNH has been rarely performed. This is partly due to the high spatial resolution required to visualise DNH and partly to the need for manual segmentation, which is operator-dependent and unfeasible in patients whose DNH is not distinguishable from the surrounding SN. An atlas-based approach would facilitate the study of this structure, as shown in some previous works^15,16^. However, while several atlases contain either a deterministic or probabilistic segmentation of SN^17–19^, DNH is not included in any existing public atlas. In a previous study, we quantified iron deposition in DNH via Quantitative Susceptibility Mapping (QSM) in PD by creating a study-specific T2*-weighted template and a probabilistic atlas from 2D DNH ROIs that were manually drawn in a group of healthy subjects^15^. However, some features of that atlas limit its generalizability and usability in studies using different contrasts or acquisition parameters. First, it relied on T2*-weighted images with limited brain coverage that could pose challenges for registration, particularly for data acquired with a different protocol. Moreover, as the region boundaries may not be clearly visible in single subject images, especially with anisotropic spatial resolution, the manual segmentation was performed on a single slice, potentially causing issues following registration, as the ROI may fall in between two adjacent slices in the target space. Finally, it was created using a relatively small number of subjects. In this work, we addressed these limitations to provide a more accurate, robust, and easy-to-register atlas. From the images of 50 healthy subjects aged 19 to 61 years old acquired *in vivo* on a 7 T scanner, we retrospectively created a 0.6 mm isotropic resolution multi-parametric template including T1-weighted, T2*-weighted, and QSM images. DNH boundaries are more clearly visible in the template than in single-subject images, allowing a more reliable manual 3D segmentation of this structure. The usage of multi-parametric information increases the versatility of the atlas, facilitating the diffeomorphic registration of different images. The atlas, which also includes the T2* maps and is provided in the MNI standard space, is meant to serve as a tool to standardise the assessment of quantitative neuroimaging findings, avoiding rater-dependent biases and allowing the study of DNH in patients whose swallow-tail sign is not visible.

## Methods

We created a multi-parametric template using diffeomorphic registrations of T1-weighted, T2*-weighted, and QSM images of 50 healthy subjects acquired on a 7 T MRI scanner. Then, eight segmentation templates were constructed from subsets of 20 randomly chosen subjects in the template space^17^. Left and right DNH were manually segmented on the T2*-weighted image and the susceptibility (*χ*) map of each segmentation template, independently by two neuroradiologists. The probabilistic atlas results from the average of the ROIs from all raters, contrasts, and segmentation templates. The pipeline employed to create the atlas is detailed in the sections below.

### Data acquisition and reconstruction

Fifty healthy volunteers (age: 31 ± 9 [19–61] years old, 21 females) were included in this study. Each subject underwent an examination on a 7 T MRI scanner (SIGNA7T, GE HealthCare) equipped with a two‐channel transmitter/32‐channel receiver head coil (Nova Medical, Wilmington, MA, USA) after giving their written informed consent to participate in the study and to the publication of their anonymized data. Data was collected in study protocols approved by the Paediatric and Area Vasta Nord Ovest sections of the regional Ethics Committee of Tuscany, Italy (CEAVNO no. 17664 - 24/06/21; CEPR no. 169/2019, 231/2021, 144/2024).

The acquisition protocol included a 3D T1-weighted sequence and a 3D multi-echo Gradient Recalled Echo (GRE). The T1-weighted image was acquired sagittally using an MPRAGE (Magnetization Prepared RApid Gradient Echo) sequence with voxel size = 0.8 × 0.8 × 0.8 mm^3^, matrix = 276 × 200 × 276, echo time TE = 3.5 ms, GRE repetition time TR_GRE_ = 7.3 ms, repetition time TR_MPRAGE_ = 3380 ms, inversion time TI = 1100 ms, delay time TD = 1600 ms, scan duration = 7'23''. The 3D multi-echo GRE was prescribed axially with whole-brain coverage using the following acquisition parameters: voxel size = 0.6 × 0.6 × 0.6 mm^3^, matrix = 360 × 360 × 280, TR = 35.2 ms, number of echoes = 5, TE_1_/ΔTE/TE_5_ = 5/5.9/28.6 ms, flip angle = 10°, BW = 41.4 kHz, ARC acceleration phase x slice = 2 × 2, compressed sensing acceleration = 2, scan duration = 4'24''. The complex signal was saved to reconstruct its magnitude and phase, for each echo.

The T2* map was computed from the magnitude data by fitting a monoexponential curve using the qmr-py toolbox (https://github.com/FiRMLAB-Pisa/qmr-py). In addition, the magnitude images of all echoes were averaged to obtain a T2*-weighted image with increased signal-to-noise ratio. Brain extraction was performed using the Brain Extraction Tool (bet)^20^ in FSL 5.0.9 (FMRIB Software Library, Oxford Centre for Functional MRI of the Brain, Oxford, UK).

QSM maps were computed from the phase of GRE images with a pipeline described in a previous work^21^, using STI Suite (MATLAB toolbox, available at https://people.eecs.berkeley.edu/~chunlei.liu/software.html from UC Berkeley, Berkeley, CA, USA). Briefly, the raw phase data were unwrapped using a laplacian algorithm^22^ and processed via V-SHARP^23^ for background field removal. Finally, the dipole inversion was performed using the iLSQR method^24,25^. Susceptibility values were referred to the average susceptibility of the whole brain.

### Template construction

Bias field correction was performed using N4 algorithm in ANTs^26^ on the T1-weighted images and the echo-averaged T2*-weighted images. Then, these images were co-registered via an affine transformation to the T2*-weighted space and then aligned to the bicommissural plane, i.e., to the anterior commissure-posterior commissure (AC-PC) space. The AC-PC alignment was performed in two steps using FSL as in the minimal Human Connectome Project structural preprocessing pipeline^27^: first, an affine transformation was computed to align each subject to the MNI space using flirt. Then, the obtained affine matrix was converted into a rigid transformation using aff2rigid which was applied to the original image in the subject space using flirt. This allowed the alignment of all subjects to the same space preserving the shape and size of their head and brain.

**Fig. 1:**
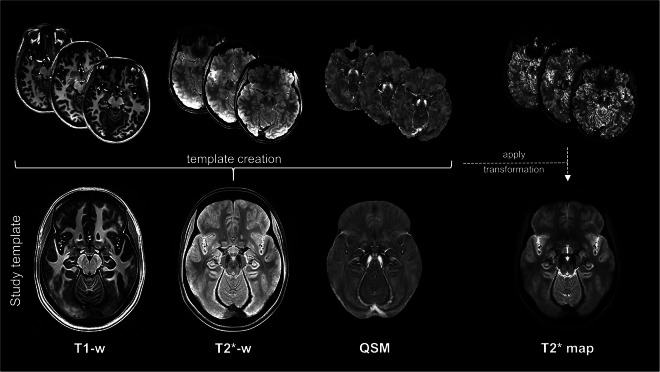
Template creation. The T1- and T2*-weighted images and the *χ* maps of all subjects (here shown, in the first row, for three exemplary subjects) were registered together to obtain the study-specific template (second row). The transformations computed to create the template were applied to the T2* map of each subject that were then combined to obtained the T2* template.

A multi-parametric template was created from the T1-weighted, T2*-weighted, and the QSM images of all subjects, using the diffeomorphic registration of the antsMultivariateTemplateConstruction2 routine in ANTs^28^. We used the SyN algorithm and a joint cross-correlation similarity metric over the three image contrasts. The other registration parameters were set as follows: gradient step size = 0.1, shrink factors = 8 × 4 × 2 × 1, maximum multi-resolution iterations = 100 × 70 × 50 × 50, smoothing factors = 3 × 2 × 1 × 0, four template-construction iterations. The T1-weighted image was assigned a weight of 0.7 while T2*-weighted and QSM images were both weighted 1, to prioritise the alignment of the subcortical structures, such as DNH, that are better delineated in T2*-sensitive images. Hence, we obtained a template of T1-, T2*-weighted images and QSM with 0.6 mm isotropic resolution in the AC-PC space, as shown in Figure 1. To obtain the corresponding quantitative T2* template, the transformations computed on the T2*-weighted images were concatenated and applied to the T2* maps of each subject to warp them to the template space. The resulting quantitative T2* template was refined using ANTs shape-update^25^ (https://github.com/ANTsX/ANTs/blob/master/Scripts/shapeupdatetotemplate.sh), and it is displayed in Figure 1.

#### DNH delineation and atlas construction

**Fig. 2:**
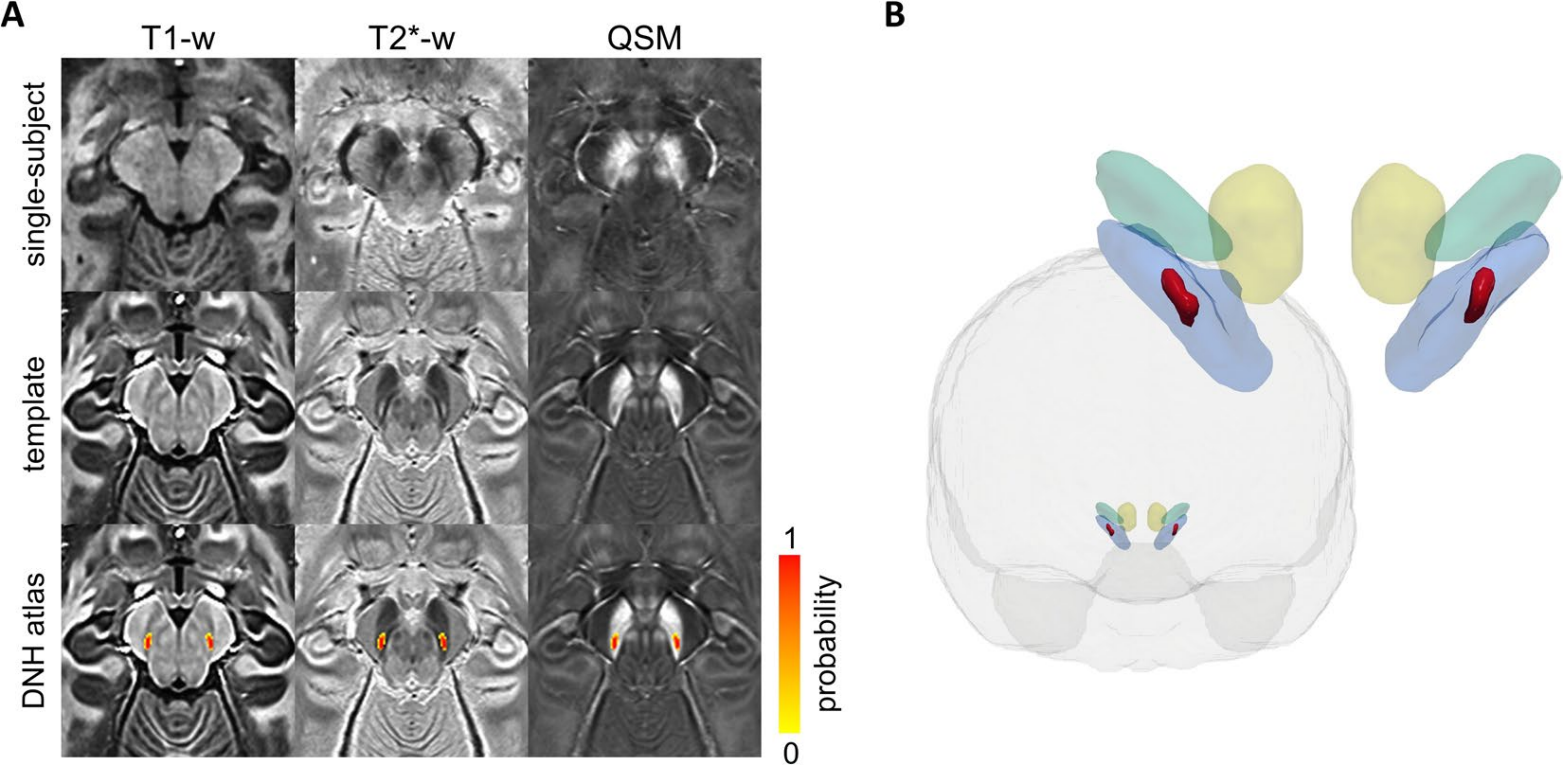
DNH atlas. (**A**) Single-subject images of a representative healthy volunteer (first row), study template (second row), and DNH probabilistic atlas overlaid onto the T1-weighted, T2*-weighted images, and the susceptibility map (third row) of the template. (**B**) 3D frontal view of the brain and the DNH ROI (red) obtained using a 0.5 probability threshold on the atlas. For anatomical reference, DNH is shown together with red nuclei (yellow), substantia nigra (blue), and subthalamic nuclei (green) from the PD25 atlas^18^ in the MNI152 2009b nonlinear asymmetric space.

The delineation of DNH in single-subject images may not be trivial due to the large variability in dimensions and appearance, so the confidence in the identification of its borders varies across subjects. However, the group averaged template yielded higher clarity in the depiction of anatomical details and the region boundaries appeared more defined and easily traceable, as shown in Figure 2A. Therefore, to generate a probabilistic atlas, we created a set of templates, which will be referred to as segmentation templates, using subsets of randomly selected subjects^17^. The subject list was scrambled and the first two groups of 20 subjects were used to create two segmentation templates by averaging them in the template space and applying ANTs shape-update routine^29^. An automatic check ensured that every subject was included in at least one segmentation template. This procedure was performed 4 times yielding a total of 8 segmentation templates.

The segmentation was performed by two expert neuroradiologists (10 and 9 years of experience) in FSLeyes. For each segmentation template and each hemisphere, each rater drew, manually and blinded from the other, two independent regions of interest (ROIs) encompassing DNH, one over the T2*-weighted image and one on the susceptibility map. DNH appears hyperintense in the T2*-weighted images of healthy subjects and enclosed between two hypointense strips of substantia nigra, whilst it appears as hypointense in QSM. The anterior boundary was defined based on the difference in the signal intensity between DNH and the remaining ventral tier of the SN pars compacta, with the former being more hyperintense in T2* and hypointense in QSM than the latter. The dorsal and ventral boundaries were defined taking into account both signal intensity and morphology of DNH, which appears as an oblique strip extending in the lateral-medial direction moving from the dorsal to the ventral part of the SN.

The probabilistic atlas for DNH was created as the percentage overlap of the ROIs drawn by both raters on all the segmentation templates (based on both T2*-weighted images and susceptibility maps, separately), that is by averaging 32 manually labelled regions for each hemisphere (given by 8 segmentation templates × 2 contrasts × 2 raters). To provide the probabilistic atlas in a standard space, the T1-weighted template was warped using ANTs to the MNI152 2009b nonlinear asymmetric space, resampled to 0.6 mm isotropic spatial resolution.

**Fig. 3:**
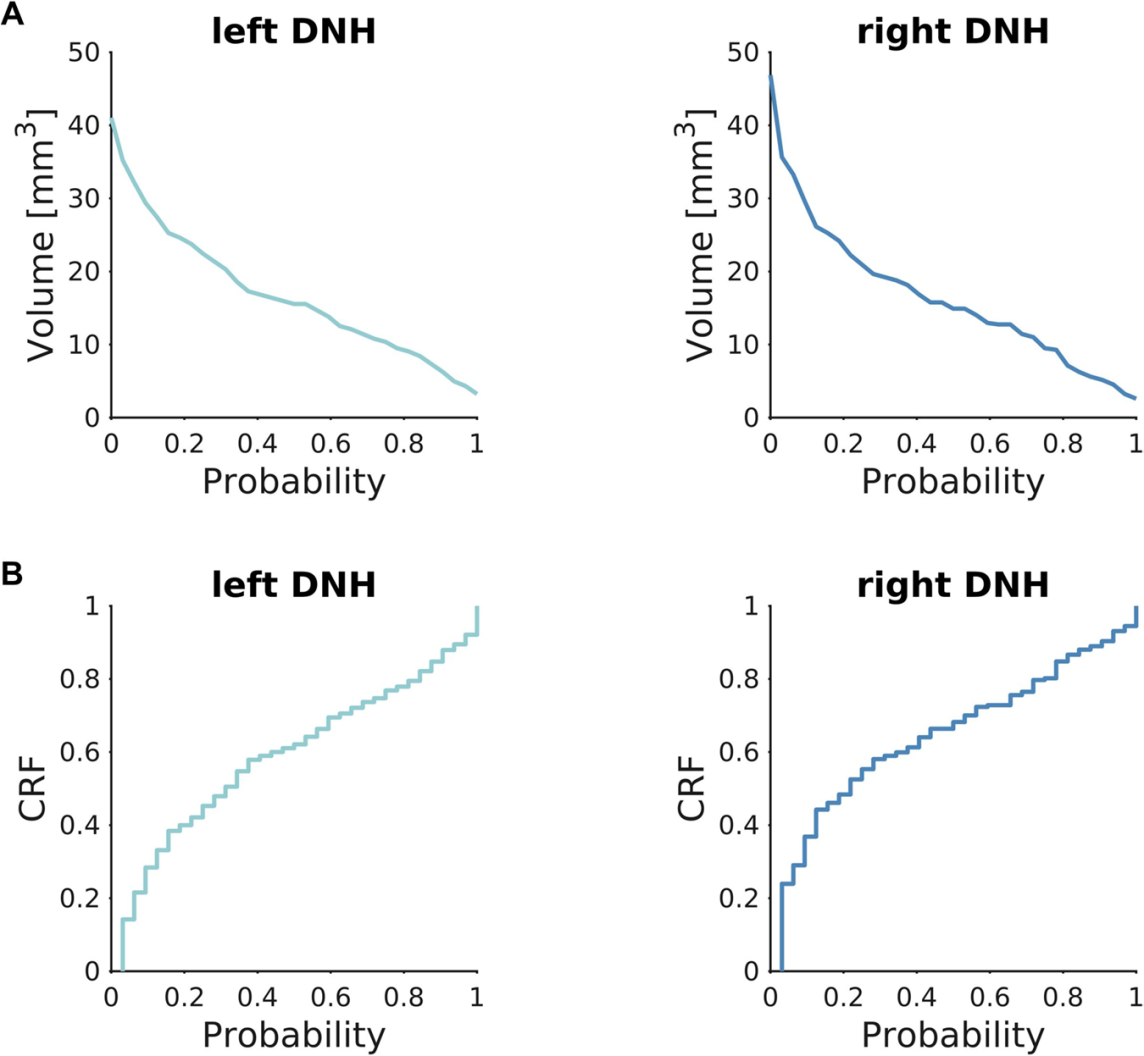
Volume and cumulative distribution of relative frequencies (CRF) of DNH ROI. (**A**) Volume of left and right DNH depending on the choice of a probability threshold. (**B**) CRF of label probability for left and right DNH. Both volume and CRF curves are computed in the study template space.

The template and the probabilistic DNH ROIs are shown in Figure 2. The volume of these ROIs depending on the probability threshold is reported in Figure 3A. The cumulative distribution of relative frequencies (CRF) of probabilities found in the probabilistic atlas provides a visual representation of the uncertainty in the delineation of DNH (Figure 3B). Despite the small dimension of DNH, the convexity of this curve is similar to the one reported for some larger structures (such as substantia nigra or subthalamic nucleus) in a well-established atlas^17^, indicating good intra- and inter-rater agreement.

## Data Records

The dataset^30^ is shared in NIfTI format on the Open Science Framework at https://osf.io/39vsd/. It contains the template, including T1- and T2*-w images, T2*, R2* and susceptibility maps, the brain mask and the probabilistic atlas of DNH in the native space and in the MNI152 2009b nonlinear asymmetric space. In addition, a script for warping the images of a subject to the template using either one or multiple image contrasts is provided. The dataset will be also available in the Lead-DBS^31^ toolbox.

## Technical Validation

The template was evaluated by characterizing DNH quantitative properties and validated by assessing the reliability of the labelling operation, through estimates of the intra- and inter-rater variability, and the agreement with manual ROIs drawn on an independent set of subjects.

### DNH quantitative characterization

We measured DNH volume, mean T2* and susceptibility, and observed their variation with age in the healthy population used to create the template. To avoid the selection of an arbitrary probability threshold, the volume was computed by integrating the voxel-wise probability of the probabilistic ROI, i.e., by summing the probability across all voxels. For the same reason, T2* and susceptibility values were obtained for each subject as a probability-weighted average. The volume was computed in the study-specific template space. The effect of age was assessed using Pearson’s correlation.

**Fig. 4:**
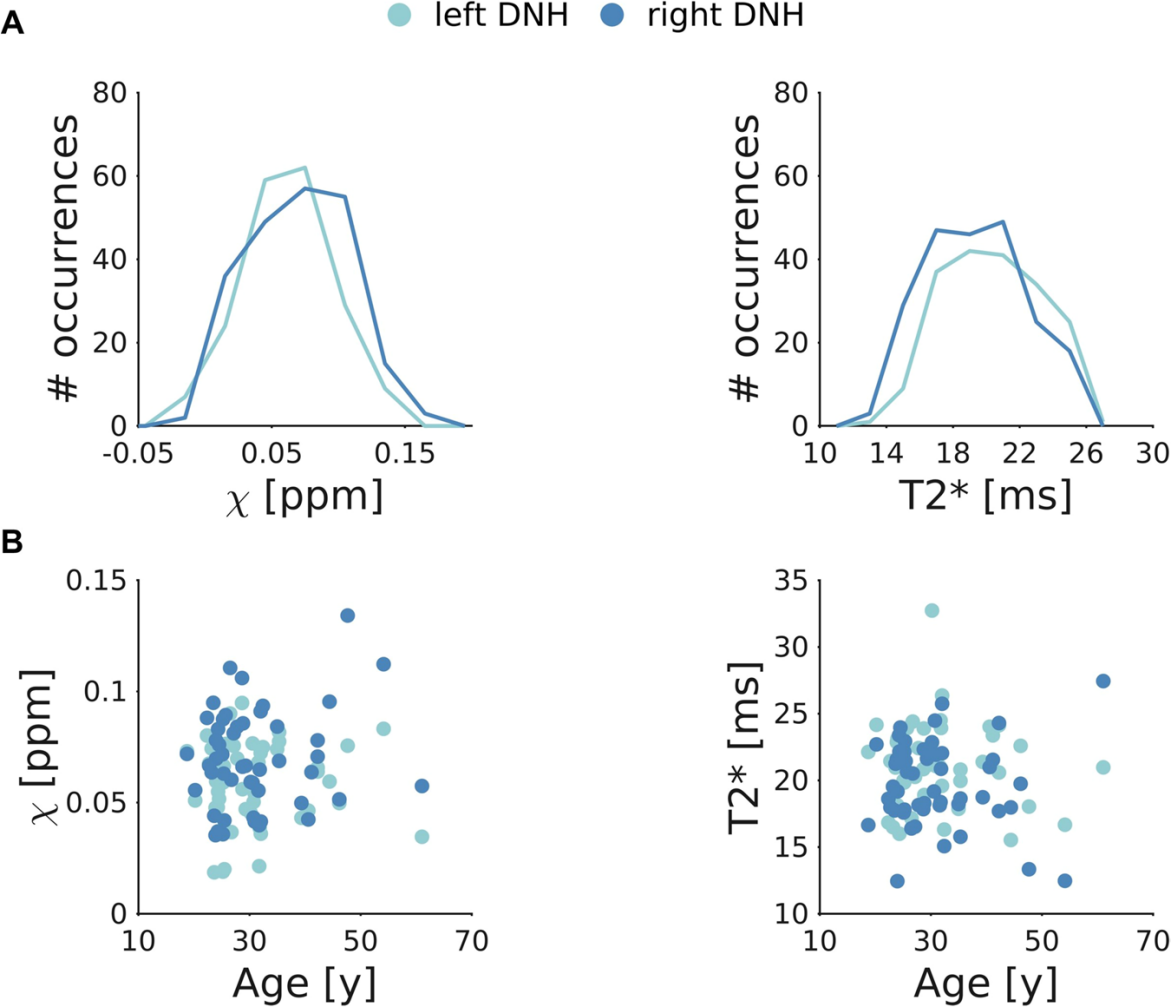
DNH characterization. Susceptibility (left column) and T2* (right column) values obtained in left and right DNH (light and dark blue, respectively). (**A**) The histogram profiles show the distribution of susceptibility and T2* found in the corresponding template within the whole probabilistic ROI. (**B**) No significant correlations with age were found in healthy subjects, for either left or right DNH.

ROI volume was very similar for left and right DNH, i.e., 17.0 mm^3^ and 16.6 mm^3^, respectively. We found a mean susceptibility of 0.059 ± 0.018 ppm and 0.070 ± 0.022 ppm and a T2* of 21.1 ± 3.1 ms and 19.7 ± 3.2 ms averaged across subjects, for left and right DNH, respectively. Figure 4A shows the susceptibility and T2* distribution within the probabilistic ROI in the template. No significant correlation with age was found for either susceptibility or T2* (Figure 4B).

### Intra- and inter-rater agreement

We used two geometric similarity metrics, i.e., Dice similarity coefficient (DSC) and Hausdorff distance (HD), to assess the consistency of the manual segmentation on the segmentation templates^17^. The DSC measures the size of the intersection between two regions normalised to their average size and was computed using the built-in Matlab function dice. The HD is the maximum distance from a point in one region to the nearest point in the other and provides an estimate of proximity between regions accounting for their shape and relative orientation. It was calculated using the ImHausdorff package in Matlab (https://github.com/joakimlindblad/ImHausdorff). Moreover, to evaluate the impact of the drawing error on the quantitative characterization of DNH, we measured the uncertainty on susceptibility and T2* computed on the templates as the standard deviation of the values obtained using different manual ROIs. All these statistics were computed by comparing ROIs drawn by the same rater on the same template contrast, by the same rater on different contrasts, and by different raters on the same contrast. The results, averaged across contrasts and raters, are reported in Table 1.

**Table 1:** Intra- and inter-rater agreement. Statistics for the assessment of the similarity (DSC and HD) of the DNH ROIs manually segmented on the segmentation templates, averaged across template contrasts and raters with the corresponding standard deviations. The standard deviation of DNH χ and T2* measured on the template provides an estimate of variability due to different segmentations.

	DSC	HD [mm]	χ std dev [ppm]	T2* std dev [ms]
Intra-rater intra-contrast	0.75 ± 0.09	1.10 ± 0.3	0.0047	0.45
Inter-rater intra-contrast	0.71 ± 0.10	1.36 ± 0.46	0.0044	0.41
Intra-rater inter-contrast	0.65 ± 0.09	1.50 ± 0.5	0.0037	0.38

Though the DSC tends to underestimate the similarity of small regions, we reported good overlap for the ROIs drawn on segmentation templates with the same contrast by the same rater (DSC > 0.7 for all contrasts and raters) or by different raters (DSC > 0.65 for both raters) and for ROIs drawn on templates with different contrasts by the same rater (DSC > 0.6 for both contrasts). Small HD was reported in all cases, ranging from 1.07 to 1.71 mm, similar to what was reported in a previous study with ROIs of other deep gray matter nuclei^17^. The average bias indicates an error lower than 0.005 ppm for susceptibility and lower than 0.45 ms for T2* when measured with different manually drawn ROIs, to be compared to an average ROI value of 0.065 ppm and 20 ms, respectively, as reported above.

### Validation on test subjects

To assess the usability and generalizability of this atlas, it was validated on an independent group of three subjects who underwent a scan session with the same acquisition protocol. DNH ROIs were manually drawn by a neuroradiologist on the T2*-w images. These were then non-linearly registered to the T2*-weighted template proposed in this study using antsRegistration and the inverse warp was applied to the DNH probabilistic atlas to transform it to the space of each subject. The geometric similarity between the probabilistic and the manually segmented ROI was evaluated via the Dice similarity coefficient and the Hausdorff distance. As the computation of these metrics requires binary images, we set a probability threshold of 0.5 for the atlas. This threshold was chosen to select voxels that are more likely to belong to DNH rather than not, resembling the decision-making process of the rater during segmentation, and as it provides an ROI with similar volume to the one obtained by integrating the voxel-wise probability of the atlas.

To evaluate the error in quantitative measurements, the average T2* and QSM values in the manual ROI were compared to the weighted averages obtained using the atlas to estimate a bias.

Comparing the probabilistic ROI in the subject space to the manually-drawn ROI, we reported an average volume difference of 3.8%. We measured DSC = 0.67 ± 0.07, HD = 1.32 ± 0.45 mm, bias on QSM = 0.0032 ppm (ranging from 0.0005 ppm to 0.009 ppm), and bias on T2* = 0.35 ms (ranging from 0.10 ms to 0.91 ms), on average across the three subjects and the two hemispheres. Both the geometric and the quantitative errors are similar to the intra- and inter-rater variability, indicating that good registration was achieved and good agreement with the manual segmentation. These findings support the generalizability of the use of the template to subjects that were not used for template creation.

### Reproducibility

**Fig. 5:**
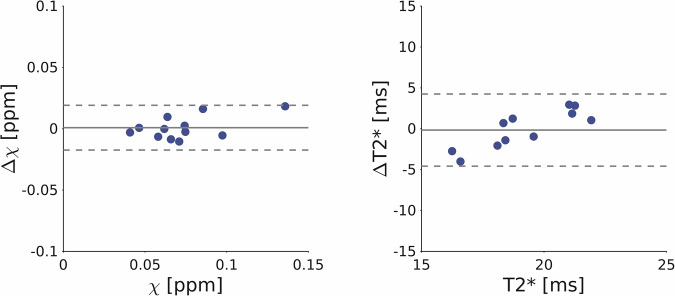
Reproducibility assessment. Bland-Altman plots of susceptibility and T2* measures in DNH for both hemispheres across repeated scans for all subjects. The grey solid line represents the average bias while the dotted lines indicate the 95% confidence interval (C.I.).

To evaluate the reproducibility of the quantification of DNH susceptibility and T2* relaxation time, six subjects included in the creation of the atlas repeated the MRI scan session within 8 months from the first one. The T2*-weighted images were then aligned to the T2*-weighted template and the inverse transformation was applied to the DNH atlas. Susceptibility and T2* were measured in the subject space as a probability-weighted average in the probabilistic DNH ROI. The Bland-Altman plot in Figure 5 shows the susceptibility and T2* differences across the two scans for all subjects and both hemispheres, together with the average bias and the 95% confidence interval (C.I.). We reported a susceptibility bias of 0.0008 ppm (C.I. = 0.018 ppm) and a T2* bias of −0.17 ms (C.I. = 4.4 ms). These results are compatible with the reproducibility found in previous works at 7 T, both in the whole brain^32^ and in subcortical nuclei^33^.

## Usage Notes

This dataset^30^ contains high resolution multi-parametric templates of the adult human brain *in vivo* and a probabilistic label of a nigral sub-structure, i.e., the DNH, partially overlapping with nigrosome 1. It aims to be a useful tool for studying DNH physiopathology and its role in neurodegenerative disorders, specifically in Parkinson’s disease and atypical parkinsonisms. The delineation of DNH on single-subject images is challenging in healthy controls and not feasible in PD patients. Therefore, the atlas can be used to facilitate the study of this region, while avoiding operator-dependent biases.

The probabilistic atlas, the T1-weighted, T2*-weighted, QSM, T2* and R2* templates are available in the MNI152 2009b standard space.

The non-linear registration to the atlas can be performed using multiple contrasts. This may increase the accuracy of the alignment, which is critical given the small size of DNH and its shape variability^34^, and integration with other multi-parametric atlases^35,36^. It is advisable to check the output of the registration in healthy subjects and establish a reliable pipeline for the specific set of acquired images, that can then be confidently applied to patients whose swallow-tail sign is not visible, since in this case the inspection of DNH registration accuracy would not be possible. An example of registration script is provided in the dataset repository^30^.

The probabilistic atlas reflects the intra- and inter-rater uncertainty in the delineation of DNH, whose ventral and dorsal boundaries are particularly challenging to identify, and the physiological variability of its shape across subjects^34^. It is possible to set a probability threshold and convert the probabilistic ROI into a binary mask. However, to avoid arbitrary choices on the threshold, the probability can be used as a weight for each voxel in the computation of quantitative properties such as average susceptibility or volume.

To provide an example of the usage of this atlas, we measured the susceptibility and T2* in DNH in a group of four patients with Parkinson’s disease using the acquisition protocol described in the Methods section. To this aim, the T2*-weighted image of each patient was bias corrected using ANTs^26^ and registered to the T2*-weighted template by computing a non-linear transformation using antsRegistration^28^. The warp was then applied to the susceptibility and T2* maps. Mean *χ* and T2* in DNH for each subject were computed as a probability-weighted average using the DNH probabilistic ROI. We reported *χ* = 0.113 ± 0.025 ppm and 0.094 ± 0.036 ppm and T2* = 17.1 ± 5.0 ms and 20.1 ± 2.8 ms in left and right DNH, respectively. We compared these values with those found in the population of 50 healthy controls that were used to create the template. The Mann-Whitney U test indicated significantly higher *χ* with respect to control subjects (p < 0.001) and lower sensitivity of T2* to pathological alterations (p = 0.21) with respect to QSM, as also suggested by a previous work^36^.

## Data Availability

Pre-processing, maps reconstruction, registrations and template creation were performed using neuroimaging toolboxes and code available online as described in the Methods sections. Statistical analysis was run in Matlab using built-in functions and the ImHausdorff package (https://github.com/joakimlindblad/ImHausdorff).
